# Two GWAS-identified variants are associated with lumbar spinal stenosis and Gasdermin-C expression in Chinese population

**DOI:** 10.1038/s41598-020-78249-7

**Published:** 2020-12-03

**Authors:** Hua Jiang, Abu Moro, Yang Liu, Jiaqi Wang, Dihua Meng, Xinli Zhan, Qingjun Wei

**Affiliations:** 1 Department of Spine and Osteopathic Surgery, The First Affiliated Hospital of Guangxi Medical University, Nanning, China; 2 Department of Orthopaedic Surgery, The First Affiliated Hospital of Guangxi Medical University, No. 6 Shuangyong Road, Nanning, 530021 China

**Keywords:** Genetic association study, Genetic markers, Musculoskeletal system

## Abstract

The aim of this study is to investigate the expression levels of genome-wide association studies (GWAS)-identified variants near Gasdermin-C (GSDMC) and its association with lumbar disc degeneration (LDD) in a Chinese population. In accordance with previously reported findings, our study involved the top 4 variants; rs6651255, rs7833174, rs4130415, and rs7816342. A total of 800 participants, 400 LDD patients and 400 controls were involved in the study. The LDD patients were divided into two mutually exclusive subgroups: subgroup 1: lumbar disc herniation; subgroup 2: lumbar spinal stenosis. Genotyping were performed using TaqMan assay, and Enzyme-Linked Immunosorbent Assay (ELISA) used to measure the plasma GSDMC levels, while quantitative reverse-transcription (qRT)-PCR and immunohistochemistry (IHC) were used to evaluate the GSDMC expression levels. Among the studied variants, there were no statistically significant differences in allelic and genotypic frequencies between LDD patients and their controls (all P > 0.05). However, the subgroup analysis revealed a significant association between rs6651255 and rs7833174 in patients with lumbar spinal stenosis (subgroup 2). Furthermore, the max-statistic test revealed that the inheritance models of two variants of lumbar spinal stenosis were represented by the recessive model. The plasma and mRNA expression levels of GSDMC were significantly higher in patients with lumbar spinal stenosis compared with the control group (P < 0.05). Furthermore, the CC genotypes of rs6651255 and rs7833174 were significantly associated with increased plasma expression levels of GSDMC in patients with lumbar spinal stenosis (P < 0.01). Two GWAS-identified variants (rs6651255 and rs7833174) near GSDMC were associated with a predisposition to lumbar spinal stenosis. GSDMC protein and mRNA expression levels may have prognostic qualities as biomarkers for the existence, occurrence or development of lumbar spinal stenosis.

## Introduction

Low back pain (LBP), a highly prevalent debilitating musculoskeletal condition is a leading cause of activity limitations and work absenteeism^1, 2^. The lifetime prevalence of LBP has been reported as over 80% and the global age-standardized prevalence of LBP estimated to be 9.4%^3^. The high prevalence of LBP makes it an enormous socioeconomic burden^4, 5^, with lumbar disc degeneration (LDD) reported as a major cause of LBP^6, 7^. LDD is a complex and multifactorial disease influenced by both genetic and environmental factors^8^. The etiology of LDD has been extensively studied, albeit incomplete understanding. Over the last few decades, it has become clear that genetic factors may play important roles in the development of LDD^9, 10^.

Many genes, such as COL9A3, ACAN, ADAMTS-5, MMP-3 and VDR, have been reported to be associated with LDD in different ethnicities^11–16^. Recently, genome-wide association study (GWAS) was merged as a powerful tool for detecting genetic contributions to LDD development. A GWAS involving 4,748 lumbar disc herniation (LDH) cases and 282,590 controls identified 37 highly correlated genetic markers associating with development of LDH at the 8q24.21 near Gasdermin-C (GSDMC) in Icelandic population^17^. The GSDMC gene encodes Gasdermin C that is involved in controlling a variety of cellular processes including cell growth, autophagy and cell death^18, 19^. It is critically important for immune system balance via regulating the autophagy, and it also acts as a tumor suppressor^20^. Moreover, two independent groups performed the meta-analysis of GWAS using European cohorts with LDD phenotypes, and reported that PTPRD and PARK2 may be the novel candidate genes for LDD^21, 22^. Many GWAS-associated variants are located in non-regulatory regions of the genome, thus, increasing the difficulty in assessing the underlying molecular mechanism of the nucleotide variation^23^. The replication of the association in different ethnic groups is important to validate the results of the GWAS^24^. Based on the existing GWAS findings, we focused on rs6651255, rs7833174, rs4130415 and rs7816342, which are the top 4 variants at 8q24.21 near GSDMC gene. To the best of our knowledge, this is the first study to investigate the relationship between of these GWAS-identified variants and LDD in a Chinese population. In this study, Enzyme-Linked Immunosorbent Assay (ELISA), quantitative reverse-transcription (qRT)-PCR and immunohistochemistry (IHC) were used to evaluate the plasma and intervertebral disc expression levels of GSDMC in LDD patients and healthy controls.

## Results

### Characteristics of subjects

The characteristics of the study and control groups are summarized in Table 1. There were no statistically significant differences in age, gender, BMI, smoking history, and alcohol consumption between the study and control groups. The median age of the control group was 50.1 years, and ranged between 20.0 and 60.0 years, while that of the case group was 49.6 years, ranging from 18.0 to 69.0 years. There was no statistically significant difference between the case and control groups (P > 0.05). The disc samples from 94 patients (32 from subgroup 1; 28 from subgroup 2; and 34 from the control group) were assigned for qRT-PCR and IHC analysis. No statistically significant differences in gender, age and BMI were observed among three groups (all P > 0.05).

**Table 1:** Characteristics of the study subjects between healthy controls and all cases including two subgroups.

Characteristics		Healthy controls	All cases^a^	Subgroup 1	Subgroup 2
Age (years)	(median, years)	50.1(20.0–60.0)	49.6 (18.0–69.0)	49.1(18.0–62.0)	50.5(24.0–69.0)
Gender	Males, n (%)	194 (48.5)	188 (47.0)	132 (46.2)	56 (49.1)
	Females, n (%)	206 (51.5)	212 (53.0)	154 (53.8)	58 (50.9)
BMI in kg/m^2^	Mean ± SD	24.33 ± 4.19	24.62 ± 8.71	24.24 ± 9.03	24.83 ± 7.23
Smoking history	n (%)	168 (42.0)	177 (44.3)	122 (42.7)	55 (48.2)
Alcohol consumption	n (%)	98 (24.5)	108 (27.0)	72 (25.2)	36 (31.6)

^a^All the cases composed of the subgroup 1 and subgroup 2.

### Case–control association analysis

The genotype frequencies of the top 4 variants at 8q24.21 locus (rs6651255, rs7833174, rs4130415 and rs7816342) are shown in Table 2. There was no significant deviation of allelic frequencies from the Hardy–Weinberg equilibrium (HWE) in control group (all P > 0.05) among the studied variants. With regards to the rs6651255 and rs7833174 variants, there were no statistically significant differences in genotypic frequency between the LDD groups and the control (P > 0.05) (Table 2), however, the subgroup analysis revealed a significant association between them and lumbar spinal stenosis (Table 3). Furthermore, the use of the max-statistic test and genetic model selection analysis revealed that the inheritance models of two variants of lumbar spinal stenosis were represented by the recessive model. Thus, it was proposed that the CC genotypes of rs6651255 and rs7833174 possibly increased the susceptibility to lumbar spinal stenosis, on the contrary, the TT genotypes of rs6651255 and rs7833174 were protective. There were no statistically significant difference between the allelic or genotypic frequencies of rs4130415 and rs7816342 between the study and control groups. Furthermore, there were no statistically significant difference in rs4130415 and rs7816342 between the 2 subgroups (all P > 0.05).

**Table 2:** Genotype frequencies of rs6651255, rs7833174, rs4130415 and rs7816342 in controls and cases.

Variants	Model	Cases (n = 400)	Controls (n = 400)	OR (95% CI)	P
**rs6651255**	**Codominant**				0.057
	TT	246 (61.50)	250 (62.63)	1	
	TC	121 (30.25)	133 (33.16)	0.92 (0.68–1.25)	
	CC	33 (8.25)	17 (4.21)	1.97 (1.07–3.63)	
	**Dominant**				0.771
	TT	246 (61.50)	250 (62.50)	1	
	CC + TC	154 (38.50)	150 (37.50)	1.04 (0.78–1.39)	
	**Recessive**				0.019
	TT + TC	367 (91.75)	383 (95.75)	1	
	CC	33 (8.25)	17 (4.25)	2.03 (1.11–3.70)	
	The P-value of global analysis: 0.0427 (MAX3) and 0.0359 (GMS)				
**rs7833174**	**Codominant**				0.444
	TT	230 (57.50)	241 (60.25)	1	
	TC	142 (35.50)	139 (34.75)	1.07 (0.80–1.44)	
	CC	28 (7.00)	20 (5.00)	1.46 (0.80–2.68)	
	**Dominant**				0.429
	TT	230 (57.50)	241 (60.25)	1	
	CC + TC	170 (42.50)	159 (39.75)	1.12 (0.85–1.49)	
	**Recessive**				0.233
	TT + TC	372 (93.00)	380 (95.00)	1	
	CC	28 (7.00)	20 (5.00)	1.43 (0.79–2.58)	
	The P-value of global analysis: 0.4187 (MAX3) and 0.3331 (GMS)				
**rs4130415**	**Codominant**				0.533
	TT	236 (59.00)	250 (62.50)	1	
	TC	139 (34.75)	130 (32.50)	0.98 (0.73–1.32)	
	CC	25 (6.25)	20 (5.00)	1.32 (0.72–2.44)	
	**Dominant**				0.311
	TT	236 (59.00)	250 (62.50)	1	
	CC + TC	164 (41.00)	150 (37.50)	1.16 (0.87–1.54)	
	**Recessive**				0.443
	TT + TC	375 (93.75)	380 (95.00)	1	
	CC	25 (6.25)	20 (5.00)	1.27 (0.69–2.32)	
	The P-value of global analysis: 0.4619 (MAX3) and 0.3244 (GMS)				
**rs7816342**	**Codominant**				0.407
	AA	225 (56.25)	237 (59.25)	1	
	AG	136 (34.00)	134 (33.50)	1.06 (0.79–1.44)	
	GG	39 (9.75)	29 (7.25)	1.42 (0.85–2.37)	
	**Dominant**				0.390
	AA	225 (56.25)	237 (59.25)	1	
	GG + AG	175 (43.75)	163 (40.75)	1.13 (0.85–1.50)	
	**Recessive**				0.205
	AA + AG	361 (90.25)	371 (92.75)	1	
	GG	39 (9.75)	29 (7.25)	1.38 (0.84–2.28)	
	The P-value of global analysis: 0.3723 (MAX3) and 0.3067 (GMS)				

*OR* odds ratio, *CI* confidence interval, *MAX3* max-statistic test, *GMS* genetic model selection.

**Table 3:** Genotype frequencies of rs6651255, rs7833174, rs4130415 and rs7816342 in controls and subgroups.

Variants	Subgroup 1 (n = 286)	Controls (n = 400)	OR (95% CI)	P	Subgroup 2 (n = 114)	Controls (n = 400)	OR (95% CI)	P
**rs6651255**								
Codominant				0.741				8.490 × 10^–7^
TT	174 (60.84)	250 (62.63)	1		72 (63.16)	250 (62.63)	1	
TC	102 (35.66)	133 (33.16)	1.10 (0.80–1.52)		19 (16.67)	133 (33.16)	0.50 (0.28–0.85)	
CC	10 (3.50)	17 (4.21)	0.85 (0.38–0.189)		23 (20.17)	17 (4.21)	4.70 (2.38–9.27)	
Dominant								
TT	174 (60.84)	250 (62.50)	1	0.659	72 (63.16)	250 (62.50)	1	0.898
CC + TC	112 (39.16)	150 (37.50)	1.07 (0.78–1.47)		42 (36.84)	150 (37.50)	0.97 (0.63–1.50)	
Recessive								
TT + TC	276 (96.50)	383 (95.75)	1	0.617	91 (79.82)	383 (95.75)	1	2.152 × 10^–6^
CC	10 (3.50)	17 (4.25)	0.81 (0.37–1.81)		23 (20.18)	17 (4.25)	5.69 (2.92–11.10)	
The P-value of global analysis: 0.8565 (MAX3) and 0.8508 (GMS)					The P-value of global analysis: 2.21 × 10^–6^ (MAX3) and 2.29 × 10^–6^ (GMS)			
**rs7833174**								
Codominant				0.222				4.198 × 10^–5^
TT	166 (58.04)	241 (60.25)	1		64 (56.14)	241 (60.25)	1	
TC	112 (39.16)	139 (34.75)	1.17 (0.85–1.61)		30 (26.32)	139 (34.75)	0.81 (0.50–1.32)	
CC	8 (2.80)	20 (5.00)	0.58 (0.25–1.35)		20 (17.54)	20 (5.00)	3.77 (1.91–7.42)	
Dominant								
TT	166 (58.04)	241 (60.75)	1	0.562	64 (56.14)	241 (60.75)	1	0.431
CC + TC	120 (41.96)	159 (39.75)	1.10 (0.80–1.49)		50 (43.86)	159 (39.75)	1.18 (0.78–1.80)	
Recessive								
TT + TC	278 (97.20)	380 (95.00)	1	0.151	94 (82.46)	380 (95.00)	1	1.032 × 10^–5^
CC	8 (2.78)	20 (5.00)	0.55 (0.24–1.26)		20 (17.54)	20 (5.00)	4.04 (2.09–7.82)	
The P-value of global analysis: 0.2905 (MAX3) and 0.9982 (GMS)					The P-value of global analysis: 2.03 × 10^–5^ (MAX3) and 2.11 × 10^–5^ (GMS)			
**rs4130415**								
Codominant				0.410				0.085
TT	166 (58.04)	250 (62.50)	1		70 ( (61.40)	250 (62.50)	1	
TC	107 (37.41)	130 (32.50)	1.07 (0.78–1.47)		32 (28.07)	130 (32.50)	0.88 (0.55–1.40)	
CC	13 (4.55)	20 (5.00)	0.97 (0.47–2.02)		12 (10.53)	20 (5.00)	2.14 (0.99–4.60)	
Dominant								
TT	166 (58.04)	250 (62.50)	1	0.239	70 (61.40)	250 (62.50)	1	0.831
CC + TC	120 (41.96)	150 (37.50)	1.20 (0.88–1.64)		44 (38.60)	150 (37.50)	1.05 (0.68–1.61)	
Recessive								
TT + TC	273 (95.45)	380 (95.00)	1	0.784	102 (89.48)	380 (95.00)	1	0.031
CC	13 (4.55)	20 (5.00)	0.90 (0.44–1.85)		12 (10.52)	20 (5.00)	2.24 (1.06–4.72)	
The P-value of global analysis: 0.4234 (MAX3) and 0.4313 (GMS)					The P-value of global analysis: 0.0656 (MAX3) and 0.0559 (GMS)			
**rs7816342**								
Codominant				0.623				0.226
AA	160 (55.94)	237 (59.25)	1		65 (57.02)	237 (59.25)	1	
AG	101 (35.32)	134 (33.50)	1.12 (0.81–1.55)		35 (30.70)	134 (33.50)	0.95 (0.60–1.51)	
GG	25 (8.74)	29 (7.25)	1.28 (0.72–2.26)		14 (12.28)	29 (7.25)	0.31 (0.17–0.58)	
Dominant								
AA	160 (55.94)	237 (59.25)	1	0.387	65 (57.02)	237 (59.25)	1	0.669
GG + AG	126 (44.06)	163 (40.75)	1.14 (0.84–1.56)		49 (42.98)	163 (40.75)	1.10 (0.72–1.67)	
Recessive								
AA + AG	261 (91.26)	371 (92.75)	1	0.475	100 (87.72)	371 (92.75)	1	0.087
GG	25 (8.74)	29 (7.25)	1.23 (0.70–2.14)		14 (12.28)	29 (7.25)	1.79 (0.91–3.52)	
The P-value of global analysis: 0.5492 (MAX3) and 0.3912 (GMS)					The P-value of global analysis: 0.1720 (MAX3) and 0.3622 (GMS)			

*Subgroup 1* patients with lumbar disc herniation, *Subgroup 2* patients with lumbar spinal stenosis, *OR* odds ratio, *CI* confidence interval, *MAX3* max-statistic test, *GMS* genetic model selection.

### Plasma GSDMC levels and LDD

**Figure 1:**
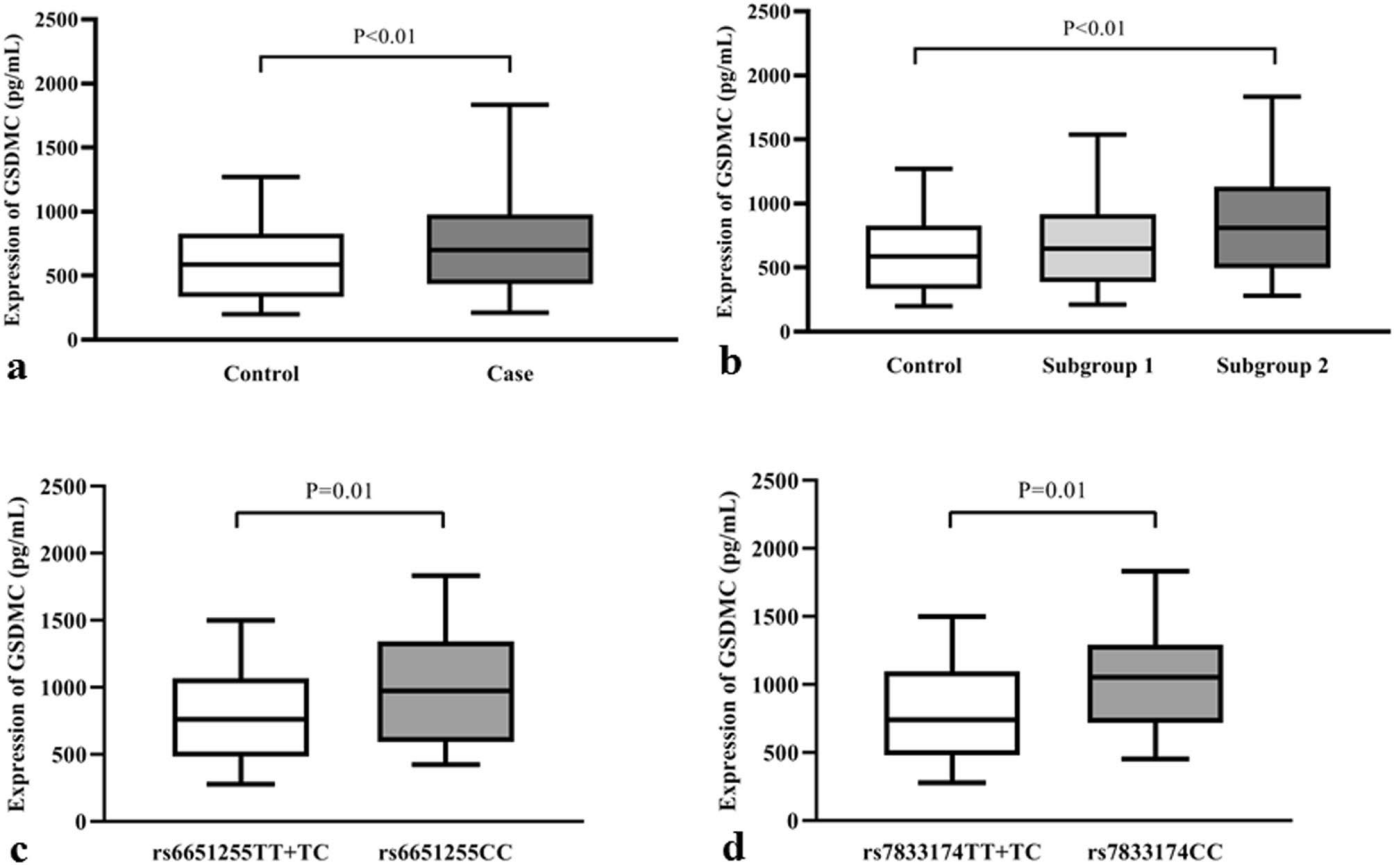
Plasma GSDMC levels were increased in lumbar spinal stenosis subgroup. (**a**) Plasma GSDMC levels were significant higher in the case group than the control group. (**b**) Plasma GSDMC levels were significant higher in the subgroup 2 than the control. (**c**) Plasma GSDMC levels were significant higher in patients with the rs6651255 CC genotype than the TT/TC genotypes. (**d**) Plasma GSDMC levels were significant higher in patients with rs7833174 CC genotype than the TT/TC genotypes. Boxplot Showing changes in the analyzed markers in the study groups and the control subjects and the median, maximum, and minimum ranges indicated.

The plasma expression levels of GSDMC for the case and control groups are shown in Fig. 1. The mean plasma GSDMC level for the control group was 592.33 ng/L (range 198.35–1270.26 ng/L), while that of the case groups was 726.77 ng/L (range 187.27–1833.20 ng/L). In case groups, the levels for subgroups 1 and 2 were 671.44 ng/L (range 187.27–1536.31 ng/L) and 837.42 ng/L (range 280.72–1833.20 ng/L) respectively. There was a statistically significant difference in plasma GSDMC levels between case and control (P < 0.01) as well as subgroup 2 and the control (P < 0.01), but not between subgroup 1 and the control (P > 0.05) (Fig. 1a,b). Thus, indicating that increased plasma GSDMC levels were significantly observed in patients with lumbar spinal stenosis. It was also observed that the plasma expression levels of GSDMC in subgroup 2 with rs6651255 or rs7833174 CC genotypes were significantly higher than those with TT + TC (all P < 0.05) (Fig. 1c,d).

### GSDMC expressions and LDD

**Figure 2:**
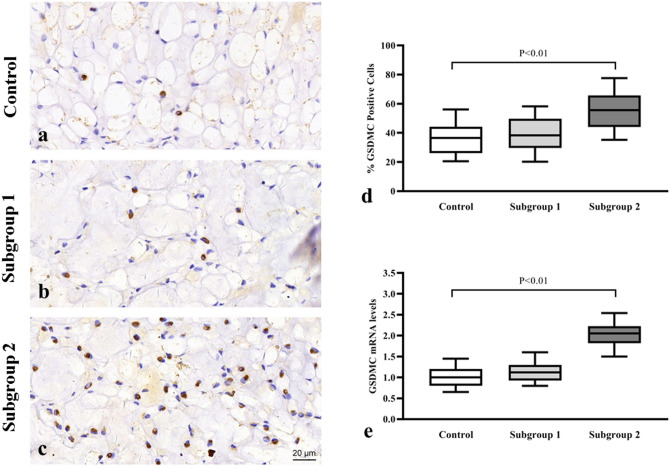
GSDMC expression levels were unregulated in the lumbar spinal stenosis (subgroup 2). (**a**–**c**) Intervertebral disc tissues harvested from the control and both LDD subgroups (Subgroup 1 and 2), respectively. (scale bar = 20 μm). (**d**) Immunohistochemistry analysis showing positive cell counting. (**a**–**d**) Expression of GSDMC was higher in subgroup 2 than the control group. (**e**) The qRT-PCR data showing the mRNA levels of GSDMC were increased in subgroup 2 compared with the control group.

The results of the qRT-PCR and IHC were consistent with our previously reported plasma findings. It was observed that the GSDMC mRNA level were increased 1.8 fold in subgroup 2 (Fig. 2e) when compared with the control, and the difference was statistically significant (P < 0.05). However, there were no statistically significant difference between the GSDMC mRNA level in subgroup 1 and control group (P > 0.05). The results of the IHC analysis also revealed that the GSDMC expressions levels were significantly higher in subgroup 2 than the control group (immunopositive cells: 62.19 ± 6.14% vs. 34.87 ± 3.34% respectively; P < 0.001) (Fig. 2a,c,d). However, the difference in expression levels between subgroup 1 and the control (Fig. 2a,b,d) was not statistically significant (immunopositive cells: 39.71 ± 4.85% vs. 34.87 ± 3.34% respectively; P = 0.072).

## Discussion

To the best of our knowledge, this is the first study to investigate the possible relationship between the top 4 GWAS-identified variants (rs6651255, rs7833174, rs4130415, rs7816342) and LDD in a Chinese population. Our results revealed that 2 of the top 4 variants, i.e. rs6651255 and rs7833174 were associated with increased susceptibility to lumbar spinal stenosis. The CC genotypes of both rs6651255 and rs7833174 were found to assert a high genetic predisposition to lumbar spinal stenosis. Furthermore, we identified significantly increased plasma GSDMC levels and related gene expressions in patients with lumbar spinal stenosis.

A recent study by Bjornsdottir et al*.* on GWAS in Icelandic population identified 37 correlated variants at 8q24.21 near GSDMC to be the risk loci for disc degeneration^17^. According to the genome-wide significance thresholds, their findings identified 4 variants among the 37 variants (rs6651255, p = 5.61 × 10^–12^; rs7833174, p = 5.97 × 10^–12^; rs4130415, p = 5.99 × 10^–12^; and rs7816342, p = 7.81 × 10^–12^) to be highly correlated with LDH. A subsequent meta-GWAS on chronic back pain involving 158,000 individuals of European ancestry further confirmed rs7833174 to be strongly associated with chronic back pain (P = 4.4 × 10^–13^)^25^. A recent study by Wu et al*.*^26^ evaluated the relationship between other GSDMC gene variations (rs4527833, rs77681114, rs4285452, rs4733741 and rs4509280) and LDH risk, and identified that rs77681114 was significantly associated with a decreased risk of LDH. However, these findings were inconsistent with our study as our results found neither the allelic nor the genotypic frequencies of these variants (rs6651255, rs7833174, rs4130415 and rs7816342) to be significantly different between our LDD group and control. Our analysis of the overall cohort of LDD patients resulted in their classification into two mutually exclusive subgroups in accordance with their presented clinical condition; i.e. disc herniation or spinal stenosis. This approach allowed for better definition and investigation into which of the relationships among the observed variants were true for all LDD patients or our predefined subgroups and how our subgroups of particular lumbar spine pathologies interacted. Interestingly, the results of our subgroup analysis of the variants rs6651255 and rs7833174, revealed statistically significant differences in both the allelic and genotypic distributions between subgroup 2 (lumbar spinal stenosis) and the control. The genetic heterogeneity among different ethnic populations may be a major explanation for different results. The previously reported studies involved subjects from European backgrounds^17, 25^, whiles the current study focused on an ethnic Chinese population. The ethnic and genetic diversity coupled with social and cultural differences may reflect in both genotypic and phenotypic heterogeneity between Chinese populations and other nationalities. Furthermore, the term LDD as a general phenotype has been used synonymously with a multitude of terminologies, such as disc herniation, spinal stenosis and spondylolisthesis. LDH happens as a result of aging and the break down that occurs within the intervertebral disc^27^. In contrast, spinal stenosis not only is caused by intervertebral disc degeneration but may also arise because of degenerative changes in the facet joints, and/or ligamentum flavum^28^. Clinically, spinal stenosis was usually defined as a specific phenotype secondary to disc herniation, which at this point represented the advanced stage of LDD. Bjornsdottir et al*.*^17^ found that these variants were associated with LDH. In this study, significant association was only observed in spinal stenosis subgroup, but not in LDH subgroup. This discrepancy could be explained by the fact that LDD has a complex and heterogeneous pathogenesis^29–31^. Therefore, from our results, we postulated that these variants may exert more influence in the advanced stages of LDD development in Chinese population.

The closest protein coding gene for rs6651255, rs7833174, rs4130415 and rs7816342 at 8q24.2 is GSDMC, and as a member of the Gasdermin superfamily, it is primarily expressed in the trachea and spleen and thought to be involved in the regulation of apoptosis and immune-related functions^18, 19, 32^. The immune system has been reported to be activated in the development of pathogenic disc degeneration, and numerous studies have confirmed the migration of immune cells into the nucleus pulpous and increased levels of inflammatory cytokines in degenerated discs^33, 34^. However, the relationship between rs6651255, rs7833174 variants and GSDMC expression has not been previously reported. In the current study, we observed the plasma levels GSDMC were significantly higher in lumbar spinal stenosis subgroup compared to the control. Correspondingly, the results of the qRT-PCR and IHC revealed elevated expression levels of GSDMC in degenerated discs. Thus, these results indicate that elevated expression levels of GSDMC to could signify an increased risk for lumbar spinal stenosis. Moreover, our investigation into whether these variants could affect plasma levels of GSDMC revealed that the CC genotypes of rs6651255 and rs7833174 were significantly associated with higher expression levels of GSDMC compared to the TT + TC genotypes. In summary, our findings identified the CC genotypes of rs6651255 and rs7833174 as a possible genetic risk factor indicator for development of LDD, and probably involved in increasing the gene expression levels of GSDMC. However, the exact molecular methodology by which it influences LDD development requires further investigations.

There are several limitations to this study. Firstly, the sample size is relatively small, hence diminishing the statistical power to detect the much smaller effects of the studied genetic variations in our subgroup analysis. Secondly, we could since only participants who consented to the study were from a single institution were involved in the study, there may be some level of the potential selection bias. Future studies in other populations, preferably multicentered with lager study population could be beneficial in confirming our findings.

In conclusion, two GWAS-identified variants near GSDMC (rs6651255 and rs7833174) may be related with a predisposition to lumbar spinal stenosis. And their CC genotypes may be significantly associated with increased plasma GSDMC expression level in patients with lumbar spinal stenosis, and thus could be useful as diagnostic or prognostic biomarkers for the existence, occurrence or as a risk factor for the development of lumbar spinal stenosis.

## Materials and methods

### Ethic statement

This study was approved by the Ethics Committee of the First Affiliated Hospital of Guangxi Medical University (2018-KY-NSFC-025). Written informed consent was obtained from all participants involved, and all procedures were performed in accordance with the guidelines of the institutional research committees and the Helsinki Declaration.

### Subjects

**Figure 3:**
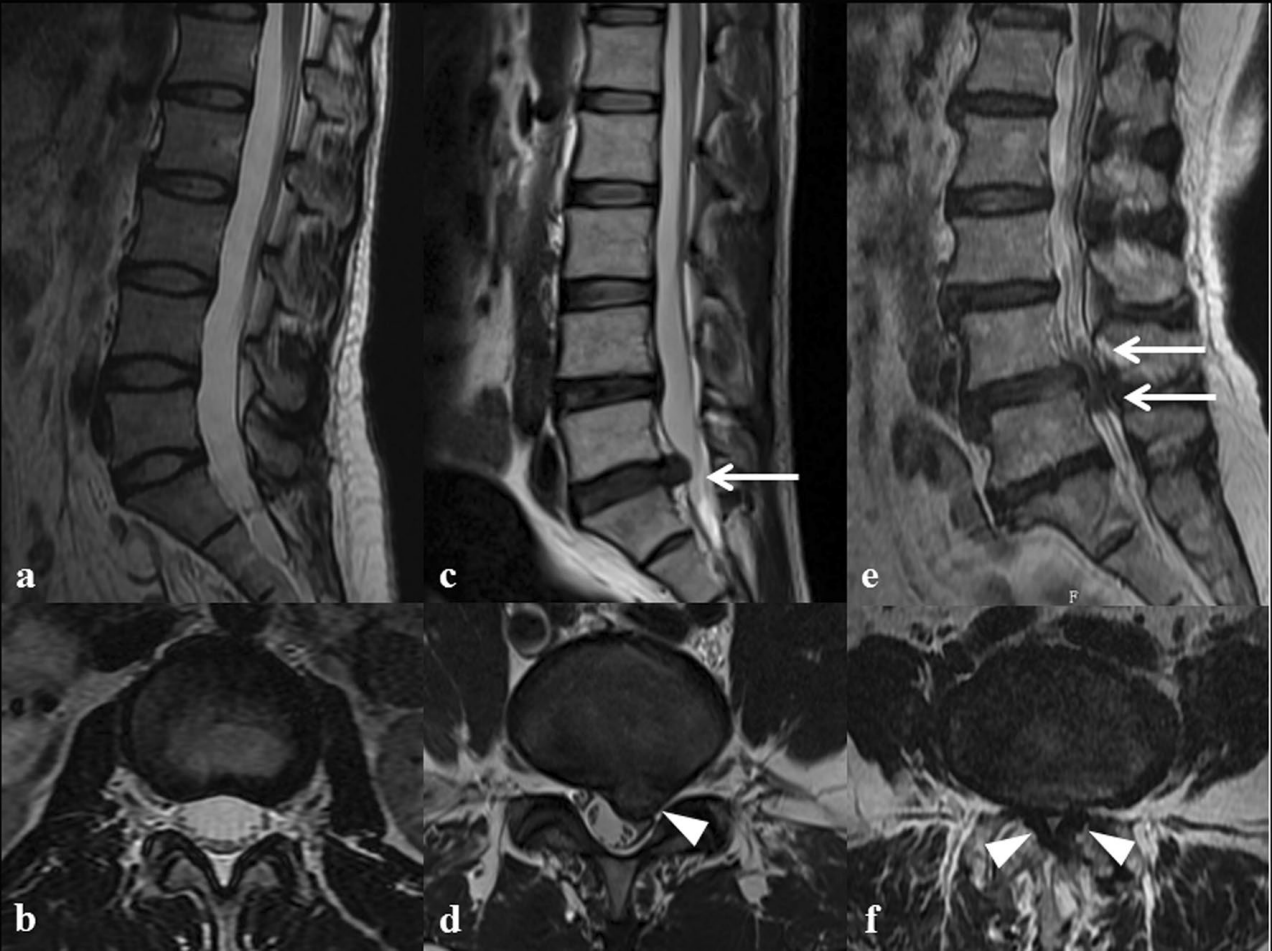
Sagittal and axial MRI features of LDD patients and normal control subjects. (**a**, **b**) Control group: Normal subjects with no disc degeneration. (**c**, **d**) Subgroup 1: patients with lumbar disc herniation; white arrows indicate the protrusion of L5/S1 herniated disc toward spinal canal. (**e**, **f**) Subgroup 2: patients with lumbar spinal stenosis; white arrows indicate narrowing of the L4-5 spinal canal and ligamentum flavum hypertrophy.

This study involved 800 participants, including 400 sporadic LDD patients (188 males, 212 females; mean age 49.6 ± 18.3 years, range 18–69 years) and 400 healthy controls (194 males, 206 females; mean age 50.2 ± 16.3 years, range 20–60 years). All participants were recruited from the Department of Spine and Osteopathic Surgery of the First Affiliated Hospital of Guangxi Medical University. All patients were examined and diagnosed by two recognized spine surgeons via physical examination and MRI. Participants were included in the study if: (1) Have history of low back pain for at least 3 months; (2) MRI scans revealed degenerative changes to the lumbar spine; (3) Have no previous history of spinal surgery or other treatments that could cause deformation of lumbar vertebrae. The control group included healthy participants with matched age and sex without history of back pain who were randomly recruited from the Physical Examination Centre of the First Affiliated Hospital of Guangxi Medical University. In accordance with the modified Pfirrmann grading system^35^, healthy subjects with Pfirrmann Grade 1 were included in the study as the control group. The LDD patients were divided into two mutually exclusive subgroups based on their MRI phenotypes (Fig. 3), with subgroup 1 involving 286 patients with lumbar disc herniation, and subgroup 2 involving 114 patients with lumbar spinal stenosis. Additionally, herniated disc tissues (n = 32), degenerative disc tissues (n = 28) and normal disc tissues (n = 34) were collected from patients with lumbar disc herniation (subgroup 1), lumbar spinal stenosis (subgroup 2) and traumatic lumbar vertebral fracture patients respectively. The patients with traumatic lumbar fracture had no previous preoperative history of low back pain, and their MRI scans revealed no disc degeneration. According to the Schneiderman’s classification^36^, there were 42 Grade 3 LDD patients (22 in subgroup 1; 20 in subgroup 2) and 18 Grade 4 LDD patients (10 in subgroup 1; 8 in subgroup 2) (Table S1). These samples were used to evaluate the GSDMC gene expression via qRT-PCR and IHC. All participants were of Zhuang ethnicity from Guangxi Zhuang Autonomous Region of China.

### DNA isolation and genotyping

In accordance with established protocols (MagNA Pure LC, Roche Applied Science, Indianapolis, IN, USA), Genomic DNA of each participant were isolated from 5 mL peripheral blood leukocytes. Qubit 3.0 fluorometer (Invitrogen) was used for DNA quantification while NanoDrop2000 spectrophotometer (Thermo Fisher Scientific, FL, USA) was used to assess DNA purity. The variants were selected based on their strong associations with LDD in GWAS^17^, and the top 4 variants among the 37 highly correlated variants i.e. rs6651255, rs7833174, rs4130415 and rs7816342 selected. The primers, probes and reaction conditions used in this study are available upon request (Table S2). TaqMan variant Genotyping Assays in an ABI 7900 sequence detection system (Applied Biosystems, Foster City, CA, USA) was used for variant genotyping, and all investigators involved blinded to the status of the subjects. Ten percent of the samples were randomly selected as duplicate quality control samples.

### ELISA detection of plasma GSDMC level

The blood samples were obtained from both LDD groups and the control group, and the plasma stored at − 80 °C after centrifugation. The serum GSDMC concentrations were measured using ELISA (Abcam, Cambridge, MA, USA) in accordance with the manufacturer’s procedural instructions. The sensitivity of the assay method was less than 2 pg/mL, and there was no cross-reactivity with other cytokines. The intra-assay and inter-assay coefficients of variation were no more than 10%, and all samples were analyzed twice.

### RNA extraction and qRT-PCR analysis

The intervertebral disc tissues were lysed in TRIzol (Invitrogen Inc, Carlsbad, CA, USA) and Rneasy Mini Kit (Qiagen, Valencia, CA, USA) used for total RNA extraction in accordance with manufacturer’s protocol. The reverse transcriptions (RT) were performed using PrimeScript RT Master Mix kit (Takara, Japan), with 1 μg total RNA used for the synthesis of the complementary DNA (cDNA) via using iScripts cDNA Synthesis kit (Quanta Biosciences, MD, USA). SYBR Green real-time PCR kit (Quanta Biosciences, MD, USA) was used to measure the relative mRNA levels, and samples normalized for GAPDH expression. All reactions were run on a real-time PCR system (Applied Biosystems) and analyzed using the comparative Ct (ΔΔCt) method (2^−ΔΔCt^ with logarithm transformation). The PCR primers used are as follows: GSDMC: Forward primer 5′-TGGAAGCAAAGACCTGACAC-3′; Reverse primer 5′-CCAAAATGATGAAGAGAATCC-3′ and GAPDH: Forward primer 5′-GACAT-GCCGCCTGGAGAAAC-3′; Reverse primer 5′-AGCCCAGGATGCCCTTTAGT-3′.

### Immunohistochemistry

The immunohistochemistry studies were performed in accordance with previously reported standard protocols^15^. The primary antibodies (Rabbit Anti-GSDMC antibody, ab254813, Abcam, Cambridge, MA, USA) were diluted at 1:50 ratio and incubated at 4 °C overnight, while the secondary antibodies (Goat Anti-Rabbit IgG, ab205718, Abcam, Cambridge, MA, USA) were incubated at room temperature for 15 min. Based on stained slides, the number of positive cells was manually counted using Olympus BX43 upright microscope.

### Statistical analysis

Differences between allelic frequencies, genotype distributions and gene expressions between cases and controls were analyzed using the standard χ^2^-analysis, with Hardy- Weinberg equilibrium used for goodness-of-fit χ^2^ test. The differences in allelic frequencies were evaluated by calculating the odd ratios and 95% confidence intervals. In order to avoid multiple comparisons by fitting three genetic models and determining the best-fitting model among them, we tested the global P value via two robust tests, max-statistic test (MAX3)^37^ and genetic model selection (GMS)^38^. For ELISA, qRT-PCR and IHC experiments, unpaired Student t-test was used for comparing the means of two groups, and one-way analysis of variance (ANOVA) with Turkey’s post-hoc test used for comparing the means of multiple groups. SPSS software Version 17.0 (Chicago, USA) and R software (Version 3.3.3, https://www.r-project.org/) with SNPassoc package^39^ were used for the data analysis, and all results considered statistically significant when P < 0.05.

### Patient consent

Obtained.

## Supplementary information

Supplementary Information.

## Data Availability

Please contact the authors for data requests.
