# Supplementary material for: Two GWAS-identified variants are associated with lumbar spinal stenosis and Gasdermin-C expression in Chinese population
Source: Sci Rep. 2020 Dec 3;10:21069. doi: 10.1038/s41598-020-78249-7 (PMC7713291; doi:10.1038/s41598-020-78249-7)
Supplement: Supplementary file 1 — Supplementary Information. [file 41598_2020_78249_MOESM1_ESM.doc]

| **Characteristics** | | **Healthy controls** | **All cases** | **Subgroup 1** | **Subgroup 2** |
| --- | --- | --- | --- | --- | --- |
| Age (years) | Median (range) | 54.2(24.8-60.0) | 53.9(23.8-69.0) | 53.5(23.8-65.0) | 54.8(34.5-69.0) |
| Gender | Male | 20 | 37 | 19 | 18 |
|  | Female | 14 | 23 | 13 | 10 |
| Location of disc tissue | L1/2 | 18 | 0 | 0 | 0 |
|  | L2/3 | 13 | 0 | 0 | 0 |
|  | L3/4 | 2 | 7 | 3 | 4 |
|  | L4/5 | 1 | 27 | 13 | 14 |
|  | L5/S1 | 0 | 26 | 16 | 10 |
| Schneiderman’s classification | 1 | 31 | 0 | 0 | 0 |
|  | 2 | 3 | 0 | 0 | 0 |
|  | 3 | 0 | 18 | 10 | 8 |
|  | 4 | 0 | 42 | 22 | 20 |
| **Table S1. Characteristics of the study subjects from whom disc tissue used in qRT-PCR and IHC**  qRT-PCR: real-time quantitative PCR; IHC: immunohistochemistry; L: lumbar; S: sacral | | | | | |

| **Variants** | **Taqman probe sequence** |
| --- | --- |
| **rs6651255** | F: ACATGGTCTTTACACATGAAGTGTGA |
|  | R: CCTCTTAGATGGCAATTCTTTGC |
|  | P-C: AGTTCAAGACCAAGGAAA |
|  | P-T: TTCAAGACTAAGGAAA |
| **rs7833174** | F: CACATAACCACCACTCTTAAAACATTTAT |
|  | R: GCTACGGACTAGACAAAGACAAAAAA |
|  | P-C: ATTCAATGACTTCAAAGTCCTTTCC |
|  | P-T: ATTCAATGACTTTAAAGTCCTTTCC |
| **rs4130415** | F: TTTCCAAACATCACTCTCTTTTCCT |
|  | R: GTTCTCAAATACAGACCCTCAAACTG |
|  | P-C: CAGTGAAAACATCG |
|  | P-T: CAGTGAAAATATCGCCGA |
| **rs7816342** | F: ACATGCCCTCACAGGAACTAAA |
|  | R: CCCTCACCATGCCTAACTGAA |
|  | P-A: TCACAGGAACTAAAATATAAAG |
|  | P-G: ACAGGAACTAAAATATGAAG |
| **Table S2.**  **Taqman probe sequence for four variants** | |
